# Sustained Firing of Model Central Auditory Neurons Yields a Discriminative Spectro-temporal Representation for Natural Sounds

**DOI:** 10.1371/journal.pcbi.1002982

**Published:** 2013-03-28

**Authors:** Michael A. Carlin, Mounya Elhilali

**Affiliations:** Department of Electrical and Computer Engineering, The Center for Language and Speech Processing, Johns Hopkins University, Baltimore, Maryland, United States of America; Indiana University, United States of America

## Abstract

The processing characteristics of neurons in the central auditory system are directly shaped by and reflect the statistics of natural acoustic environments, but the principles that govern the relationship between natural sound ensembles and observed responses in neurophysiological studies remain unclear. In particular, accumulating evidence suggests the presence of a code based on sustained neural firing rates, where central auditory neurons exhibit strong, persistent responses to their preferred stimuli. Such a strategy can indicate the presence of ongoing sounds, is involved in parsing complex auditory scenes, and may play a role in matching neural dynamics to varying time scales in acoustic signals. In this paper, we describe a computational framework for exploring the influence of a code based on sustained firing rates on the shape of the spectro-temporal receptive field (STRF), a linear kernel that maps a spectro-temporal acoustic stimulus to the instantaneous firing rate of a central auditory neuron. We demonstrate the emergence of richly structured STRFs that capture the structure of natural sounds over a wide range of timescales, and show how the emergent ensembles resemble those commonly reported in physiological studies. Furthermore, we compare ensembles that optimize a sustained firing code with one that optimizes a sparse code, another widely considered coding strategy, and suggest how the resulting population responses are not mutually exclusive. Finally, we demonstrate how the emergent ensembles contour the high-energy spectro-temporal modulations of natural sounds, forming a discriminative representation that captures the full range of modulation statistics that characterize natural sound ensembles. These findings have direct implications for our understanding of how sensory systems encode the informative components of natural stimuli and potentially facilitate multi-sensory integration.

## Author Summary

We explore a fundamental question with regard to the representation of sound in the auditory system, namely: what are the coding strategies that underlie observed neurophysiological responses in central auditory areas? There has been debate in recent years as to whether neural ensembles explicitly minimize their propensity to fire (the so-called sparse coding hypothesis) or whether neurons exhibit strong, sustained firing rates when processing their preferred stimuli. Using computational modeling, we directly confront issues raised in this debate, and our results suggest that not only does a sustained firing strategy yield a sparse representation of sound, but the principle yields emergent neural ensembles that capture the rich structural variations present in natural stimuli. In particular, spectro-temporal receptive fields (STRFs) have been widely used to characterize the processing mechanisms of central auditory neurons and have revealed much about the nature of sound processing in central auditory areas. In our paper, we demonstrate how neurons that maximize a sustained firing objective yield STRFs akin to those commonly measured in physiological studies, capturing a wide range of aspects of natural sounds over a variety of timescales, suggesting that such a coding strategy underlies observed neural responses.

## Introduction

It is widely believed that sensory representations are optimized to process the stimuli to which they are exposed in natural environments [1]. Of particular interest is understanding the computational principles that underlie the generation of observed neural firing patterns. A popular hypothesis explored in recent years assumes that neural populations optimize a sparse code. This means that at any given time, only a small subset of a neural population fires to encode a given stimulus [2]. Such a representation is attractive for reasons of coding efficiency (see, e.g., [3]) and conservation of physiological resources [4]. The sparse coding hypothesis has enjoyed particular success in studies of vision (e.g., [5], [6]), and has also been supported more recently by both neurophysiological [7], [8] and computational studies [9]–[11] of the auditory system.

However, it has also been observed that some central auditory neurons, when driven by their preferred stimuli, exhibit *sustained* firing rates. Measuring from auditory thalamus and primary auditory cortex, Wang *et al.* observed that sustained responses were not simply phase-locked to the fast dynamics of the stimulus, suggesting that this rate-based code represented a meaningful, non-isomorphic transformation of the stimulus [12], [13]. Indeed, such a code is particularly important for audition since it directly addresses the issue of how to indicate the continued presence of a sound in a complex acoustic environment. Results from Petkov *et al.* have also illustrated how sustained responses play a role in auditory scene analysis, forming part of the neural basis for the perceptual restoration of foreground sounds against a cluttered background [14]. Moreover, Wang has argued that a rate-based representation is critical for matching fast temporal modulations present in natural sounds to slower rates found in higher cortical areas [15]. Slower dynamics in acoustic signals are believed to be the main carrier of information in speech and music [16]; are commensurate with temporal dynamics of stream formation and auditory grouping [17]; and may play an important role in multi-modal sensory integration [15]. Related computational studies in vision have suggested how this principle may underlie the shapes of simple and complex cell receptive fields in primary visual cortex [18], [19]. Importantly, a sustained firing rate, i.e., one that is persistent and therefore slowly changing over time, is related to slow feature analysis, a well-known method for extracting invariances from sensory signals [20] (see Discussion). To the best of our knowledge, however, there are no computational studies that explicitly consider the implications of a sustained firing-based code in central auditory areas.

At first glance, the two coding schemes are seemingly at odds: on the one hand a sparse code seeks to minimize the activity of a neural population whereas a sustained firing-based code requires that neural responses persist over time but still form an efficient representation of the stimulus. However, it appears that central auditory responses can strike a balance between the two strategies, with a large, transient population response at the onset of a sound, and a sparse subset of preferentially driven neurons exhibiting a strong, sustained response throughout the sound's duration [15], [21]. This picture suggests a mechanism for detecting and tracking target sounds in noisy acoustic environments and for generating a persistent signal that facilitates a stable perceptual representation. From a computational perspective, a better understanding of these mechanisms can inform models of auditory scene analysis as well as signal processing schemes for hearing prosthetics and automated sound processing systems.

**Figure 1:**
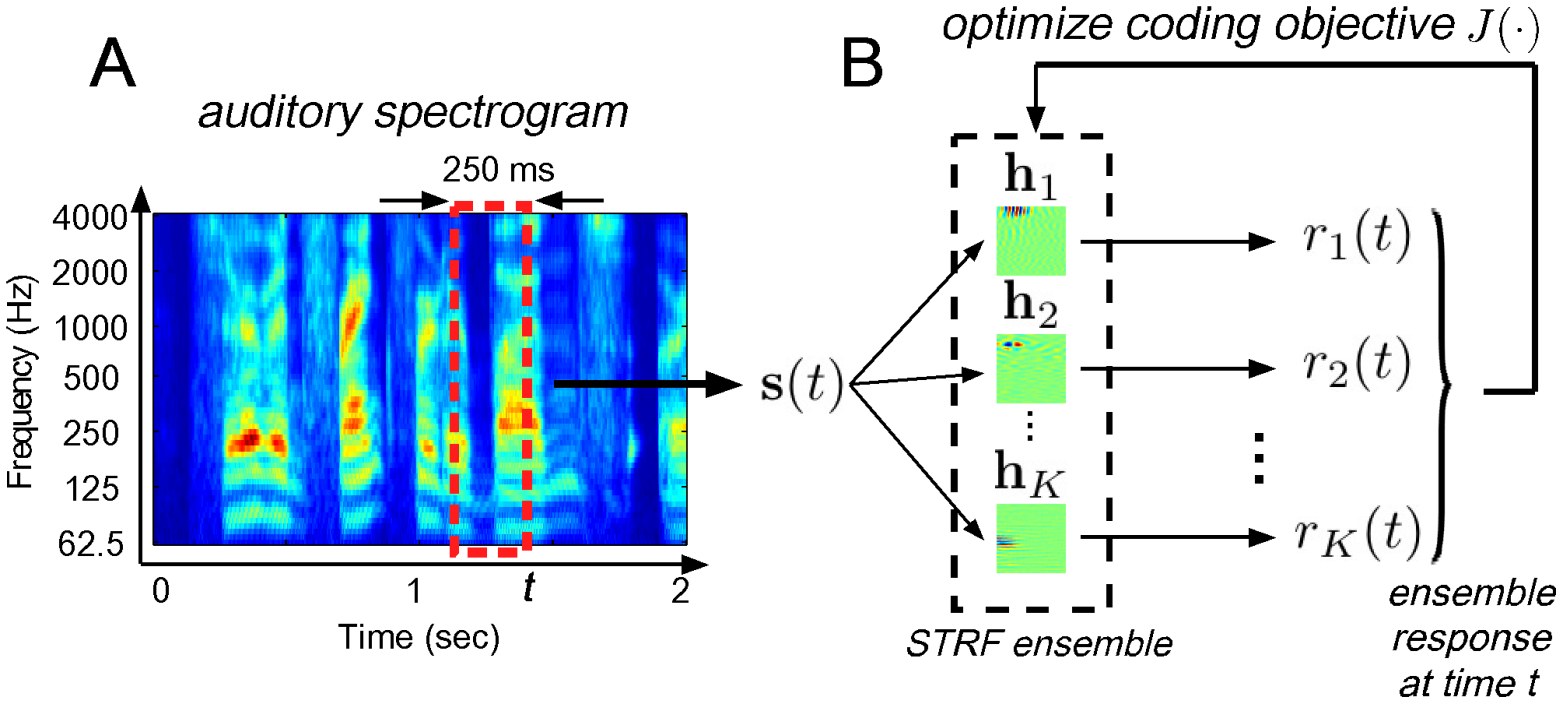
Schematic of the proposed framework. Panel (A) shows an example of an auditory spectrogram for the speech utterance “serve on frankfurter buns…” whereas panel (B) illustrates how spectro-temporal patches are mapped to an ensemble of instantaneous neural firing rates.

A general computational approach for exploring the effects of particular coding strategies in sensory systems is based on optimizing a statistical objective criterion that quantifies the principle governing the transformation between stimulus and internal representation. Upon convergence, one then compares the emergent representation to known properties of the sensory system being studied [1]. Here, we apply this framework to explore how optimizing a sustained firing criterion influences the shapes of model auditory spectro-temporal receptive fields (STRFs) when processing natural sounds, and we compare the emergent ensembles to those obtained by optimizing a sparse coding objective. STRFs describe the linear mapping between a spectro-temporal stimulus and an instantaneous firing rate [22], and have proven useful not only for describing basic processing aspects of auditory neurons [23], [24], but also for shedding light on the nature of task-driven plasticity [25]. Figure 1 illustrates how a spectro-temporal stimulus is mapped to a set of instantaneous neural firing rates, whose ensemble response according to a desired coding strategy directly shapes the mapping.

In this paper, we show how this framework allows us to not only explore how the timescales of natural sounds are captured by and reflected in an emergent sensory representation, but reveal key similarities between choice of a sustained versus sparse code. Moreover, we demonstrate how a sustained firing-based code suggests a mechanism for an emergent discriminative representation for ensembles of natural stimuli.

## Results

We defined a sustained neural response as one where firing rate energy changes relatively slowly and is consequently highly *correlated* over time. In particular, we were interested in the characteristics of ensembles of model STRFs 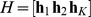 that promoted sustained responses over a specified time interval 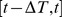. Denoting the response of the 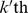 neuron as 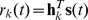, where 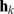 is the STRF and 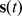 is a spectro-temporal stimulus, we quantified this principle using the following objective function:
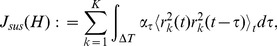
(1)where 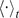 denotes time average. Observe that 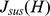 represents the sum of correlations between signal energies of the 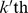 neuron over a time interval defined by 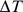 across an ensemble of 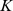 neurons. If a neuron yielded a sustained response, then each of the 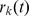 would vary smoothly over the specified interval and we expect 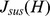 to be large. Moreover, choice of the correlation interval 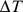 allowed us to directly explore the effect of different timescales on the ensembles 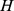 that *maximized* Eq. 1. Finally, the weights 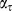 were chosen to be linearly decaying for 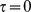 to 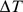, reflecting the intuition that recent activity of a neuron likely has more influence on the current output than the past. Note that these weights could be adapted to specifically model, for example, positive- or negative-monotonic sustained responses observed in physiological studies [13]. Full details of the optimization procedure can be found in Methods.

Alternatively, we explored an objective function that promoted sparsity. A natural way to induce sparsity in a population code is by enforcing a population response whose firing rate distribution is highly peaked near zero (representing frequent *weak* responses), but has long tails (representing infrequent *large* responses), i.e., a distribution with high *kurtosis* [26]. We quantified the sparsity of a population code using sample kurtosis:
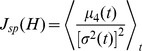
(2)where 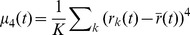 is the fourth central moment at time 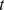, 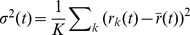 is the population variance at time 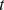, and 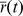 is the population mean at time 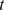.

For both 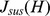 and 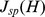, the basic problem was to find an ensemble of STRFs that *maximized* the respective objective function subject to constraints that (1) bounded the amplitude of the filter responses and (2) minimized redundancy among the learned ensemble. This was achieved by enforcing the responses have unit variance and be mutually uncorrelated, i.e., 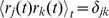 where 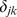 is the Kroenecker delta function (see Methods); we refer to these as *response* constraints. These constraints ensured that the responses had a bounded magnitude and that the STRFs did not all converge to the same solution.

### Emergence of richly structured STRFs

We optimized both the sustained objective 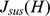 and sparsity objective 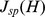 using an ensemble of natural stimuli comprising speech, animal vocalizations, and ambient outdoor sounds. Each ensemble of 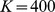 filters was initialized at random using zero-mean, unit variance Gaussian noise, and each STRF covered from 0–250 ms in time and 62.5–4000 Hz along the tonotopic axis.

**Figure 2:**
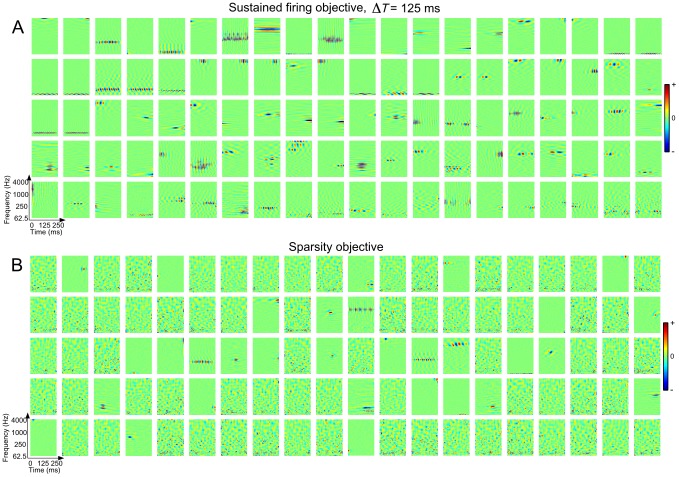
Examples of emergent STRFs. Shown are STRFs learned by optimizing (A) the sustained objective function 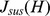 for 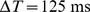 and (B) the sparsity objective function 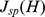. The examples shown here were drawn at random from ensembles of 400 neurons. The sustained STRFs are shown in order of decreasing contribution to the overall objective function whereas the sparse STRFs are shown randomly ordered. Each spectro-temporal patch spans 0–250 ms in time and 62.5–4000 Hz in frequency. For these examples the dynamic range of the STRFs was compressed using a 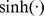 nonlinearity.

For the sustained objective, we considered a wide range of correlation intervals from very brief (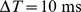) to very long (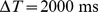). Examples of emergent STRFs for 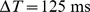 are shown in Figure 2A. For the spectro-temporal patches shown, red and blue colors indicate that the presence of energy in a particular spectro-temporal region yields excitatory and inhibitory responses, respectively. We observe a variety of STRFs that are highly localized, sensitive to narrowband spectral and temporal events, oriented, and some that are seemingly noise-like and not convergent to any particularly interesting shape. Importantly, such observations about these basic STRF classes align with those made in a number of previous physiological studies (see, e.g., [23], [24], [27]). Moreover, coverage of the STRFs appears to span the full time-frequency space. These results suggest that the sustained firing objective may underlie part of the coding strategy used by central auditory neurons.

Shown in Figure 2B are examples of emergent STRFs obtained by optimizing the sparsity objective. Indeed, this particular objective yields STRFs that are highly localized and sparsely distributed, with sensitivity to bandlimited spectral and temporal events. While both objective criteria yield noisy STRFs, it is clear that the sparse ensemble is much more noisy, with a less extensive coverage of the basic sound classes as observed with the sustained ensemble.

### Ensemble diversity varies smoothly with 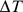


Since the information-bearing components of natural sounds vary concurrently across multiple timescales, it was expected that the structure of STRFs learned under the sustained objective would vary with the correlation interval 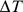. Indeed, inspection of the sustained ensembles for a range of 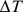 suggested the presence of a number of latent classes whose membership varied smoothly from short to long correlation intervals. To quantify variations in population diversity over ecologically relevant timescales, we performed unsupervised clustering of the emergent STRFs and studied how class membership changed with objective function and correlation interval.

We pooled STRFs from the sparse ensemble and from the sustained ensembles for 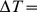
10, 25, 50, 125, 250, 500, 1000, and 2000 ms, yielding a total of 3600 STRFs. We then applied normalized spectral clustering to discover latent classes among the pooled STRFs. In general, spectral clustering algorithms require an affinity matrix that specifies pairwise similarities between the objects being clustered. Viewing this affinity matrix as an undirected graph, spectral clustering finds a partition of the graph into groups whose elements have common similarity with one another. A natural measure of similarity between STRFs can be derived from the two-dimensional cross-correlation between pairs of spectro-temporal patches. Such a measure is similar to that considered by Woolley *et al.* [28] and is desirable since it does not depend on subjective choice of spectro-temporal features to use for clustering. In this work, we defined the measure of similarity between pairs of STRFs as the *absolute value* of the maximum value of the two-dimensional cross-correlation matrix; we used absolute value since we wished to group similar STRFs regardless of whether they were excitatory or inhibitory. Furthermore, as the STRFs tended to be distributed with a variety of phases in the input space, we considered cross-correlations for arbitrary time-frequency shifts (see Methods for details).

**Figure 3:**
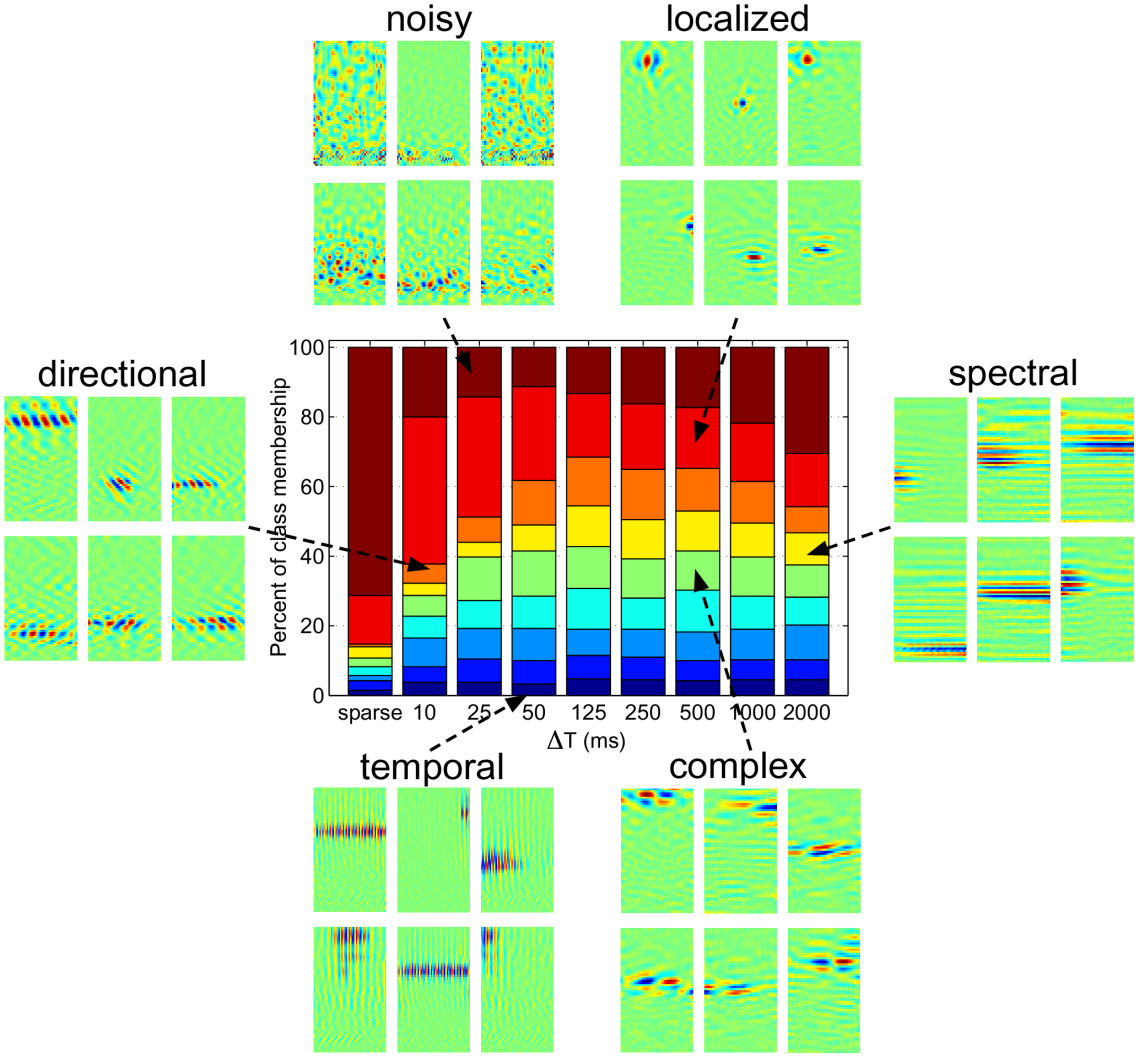
Spectral clustering results. Shown are nine clusters obtained by pooling STRFs from the sparse as well as sustained ensembles for 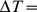
10, 25, 50, 125, 250, 500, 1000, and 2500 ms. Shown in the center is a stacked bar chart where segment color corresponds to class label and segment width is proportional to the number of STRFs assigned to a particular class in a given ensemble. The surrounding panels show examples of STRFs drawn from six illustrative classes, namely, *noisy*, *localized*, *spectral*, *complex*, *temporal*, and *directional*.

Results obtained using normalized spectral clustering of the emergent ensembles into nine classes are shown in Figure 3. In the center panel of the figure, a stacked bar chart illustrates the the percentage of STRFs at a particular 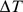 assigned to one of nine classes. Different segment colors correspond to each of the nine classes, and segment width is proportional to the number of STRFs assigned to that class. Surrounding the bar chart are examples from six classes that best illustrate how diversity varies with 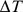, namely *noisy*, *localized*, *spectral*, *complex*, *temporal*, and *directional* classes. These labels are qualitative descriptors of each class and not quantitative assessments of the time-frequency characteristics of each category.

Inspection of the cluster groupings reveal rich structural variations over a wide range of correlation intervals. In particular, the STRFs labeled according to the *noisy* class are found to dominate the sparse ensemble, with a large presence in the sustained ensemble for 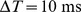. Membership in this class drops for 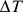 between 10 and 125 ms, and begins to increase at 125 ms. We also observe that short correlation intervals (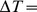
10, 25, and 50 ms) have a large concentration of *localized* STRFs, with membership dropping with increasing 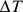. While the *temporal* class holds relatively steady across the sustained ensembles, we find that membership in the *directional*, *complex*, and *spectral* classes varied smoothly across 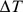. In general, we find that ensemble diversity is maximized for 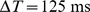 (max. entropy of 3.08 bits), but the overall trends suggest rich ensemble structure between 10 and 250 ms, which is notably in the range of the timescales of natural sounds [29], . This is further supported by the increasing presence of *noisy* STRFs for large correlation intervals (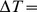
1000 and 2000 ms).

In addition to studying structural variations in the *shapes* of the emergent STRFs, it is also of interest to examine the structure of the STRF *outputs* in response to natural sounds. In particular, we sought to address the extent to which enforcing sustained responses does indeed yield responses that persist over time. We defined the 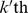 neuron to be significantly “active” when its firing rate 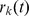 exceeded 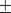
1 standard deviation over time. While this is not meant to be a precise measure of a neuron's activation (since, for instance, the firing rate is not used to modulate a Poisson spike generation process), such a measure nevertheless quantifies and characterizes a strong versus weak ensemble response to natural stimuli.

**Figure 4:**
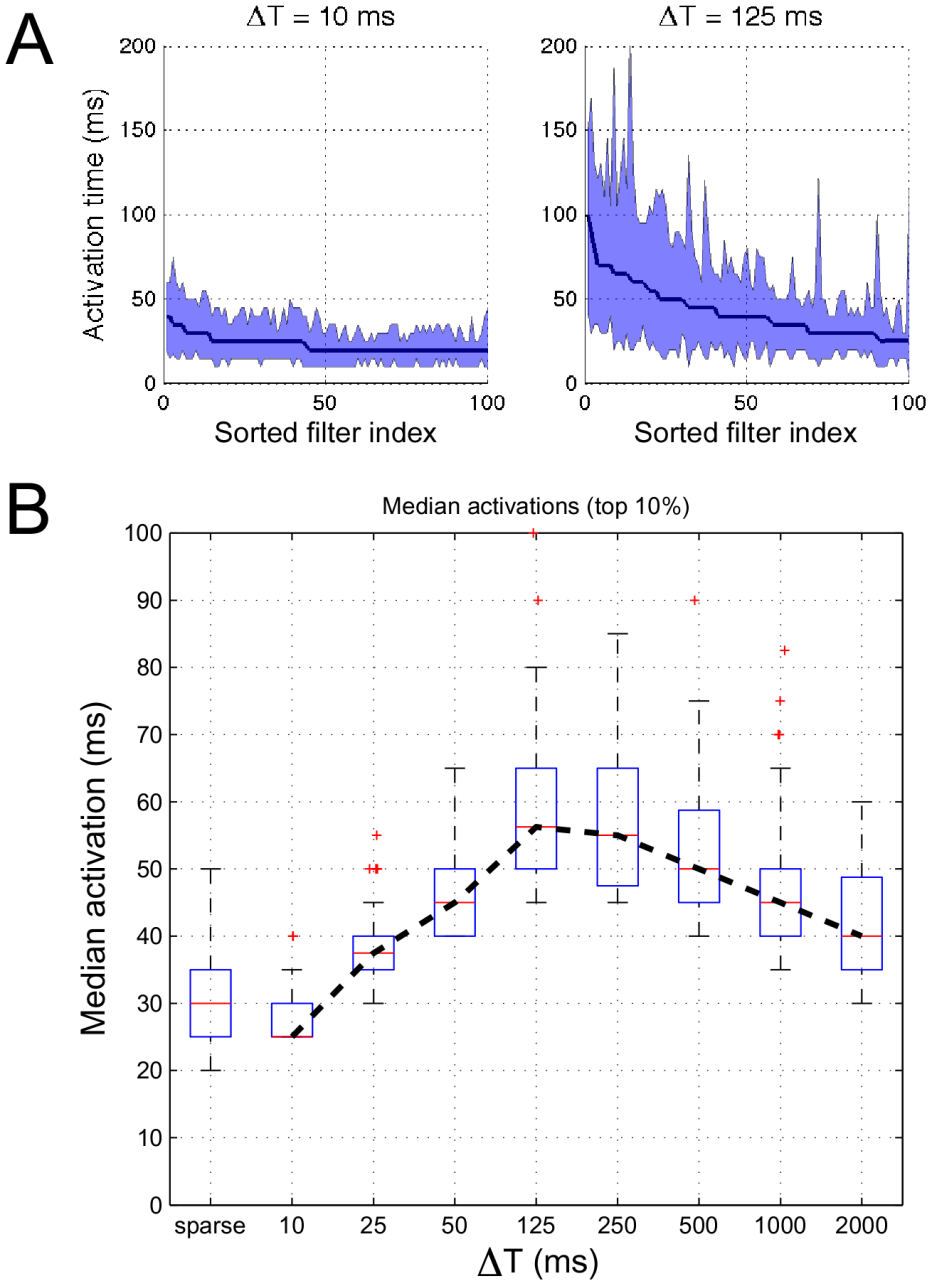
Analysis of the temporal activations of emergent ensembles. Panel (A) shows the median activation time of individual neurons (solid lines, sorted in decreasing order) for 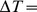
10 and 125 ms, respectively, for STRFs that optimize the sustained objective function. The shaded region illustrates the corresponding interquartile range. Panel (B) shows the distributions (as boxplots) of median activation times of the top 10% “most persistent” neurons for sparse and sustained ensembles for increasing 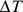.

Shown in Figure 4A are the distribution of activation times for individual neurons for ensembles of 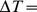
10 and 125 ms in response to a held-out set of natural stimuli. The neurons are shown sorted according to decreasing median activation time, and the interquartile ranges of activation time are indicated by the shaded regions. We observed that the most diversity in median activation times across ensembles occurred in approximately the top 10% of the *most persistent* neurons. To summarize these observations, we considered the distribution of median activation times of the top 10% of neurons with most persistent responses (i.e., the top 40 neurons); these distributions are illustrated as boxplots in Figure 4B.

As noted previously with the clustering results, shorter 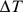 values favor mostly localized and noisy STRFs and consequently it was expected that activations would be brief. Interestingly, however, we observe that with increasing 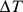, median activations peak between 50 and 500 ms and fall off for large 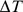 despite the STRFs being optimized to promote sustained responses over long intervals. This overall trend aligns with the previous clustering results that demonstrate how population diversity is maximized over intervals corresponding to timescales that predominate natural stimuli. The STRFs corresponding to the top 10% most persistent responses for 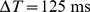 are shown in Supplementary Figure 1, and we find that they generally have a spectral tuning, but are fairly narrowband and localized.

Additionally, we considered the responses of the top 40 most persistent responses obtained using the sparsity objective function; the distribution of median activations is in the first column of Figure 4B. We find that the sparse ensemble yields responses most similar to those for short 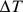.

### Comparison of emergent sustained ensembles to physiology

How do the emergent STRFs learned under the sustained firing objective compare to those observed in physiological studies? Broadly speaking, we find that the emergent STRFs share many of the trends with biological receptive fields typically observed in animal models. We explored this issue by comparing our model ensembles with a set of 1586 STRFs recorded from awake, non-behaving ferret primary auditory cortex using TORC [31] and speech stimuli [27], [32] (see Methods for more details). Where applicable, we also compared our results with reported results from anesthetized ferrets by Depireux *et al.* [23] and cats by Miller *et al.* [24] in the literature.

**Figure 5:**
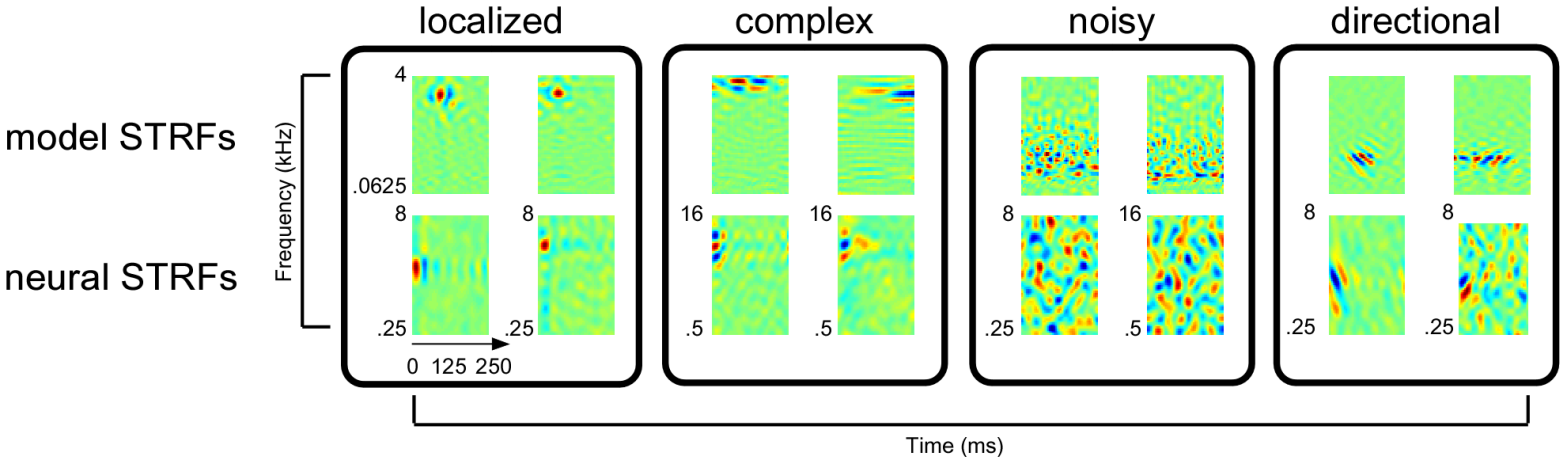
Comparison of emergent STRFs learned according to the sustained objective function with examples estimated from ferret auditory cortex.

Illustrative examples of the types of STRFs found in the neural data are shown in Figure 5. In particular, we find neural STRFs that are qualitatively similar those found in the *localized*, *complex*, *noisy*, and *directional* clusters shown earlier in Figure 3. Because the temporal and spectral sampling rates used in our model are higher than those used in the physiological data, we did not find good matches with the *temporal* and *spectral* classes.

**Figure 6:**
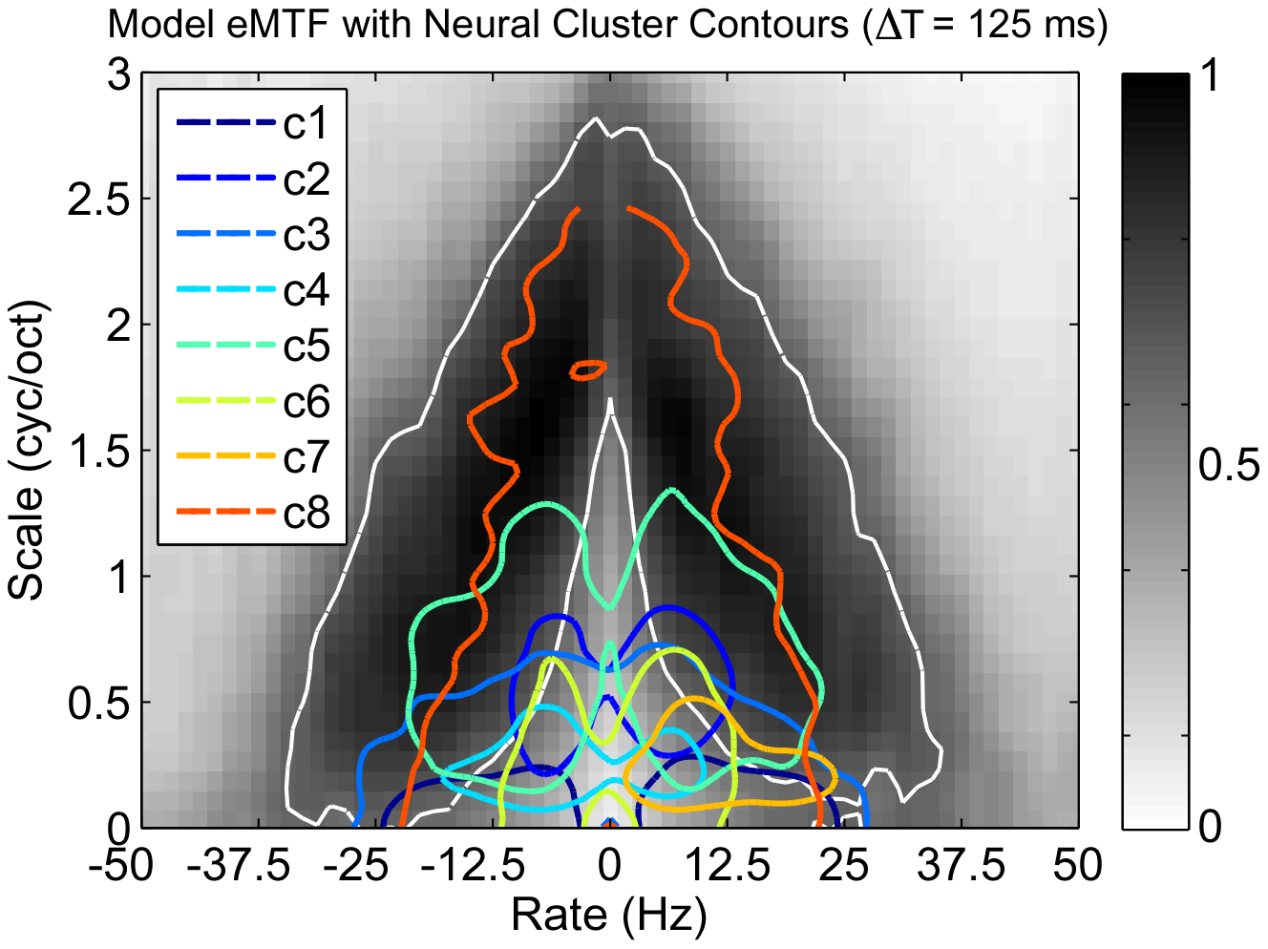
Cluster analysis of neural STRFs. Illustration of the overlap between the eMTFs of neural STRF clusters and that of the response-constrained sustained objective model STRFs; class 9 comprised mostly noisy STRFs with an exceedingly broad eMTF and its contour is omitted here for clarity. The white contour corresponds to the model eMTF at the 65% level.

To visualize the overlap between the spectro-temporal modulation coverage of the neural and model STRFs, we used the ensemble modulation transfer function (eMTF). The eMTF is derived by averaging the magnitude of the 2D Fourier Transform of each neuron in a given ensemble, and jointly characterizes modulations in time (rate, in Hz) and in frequency (scale, in cyc/oct). We first applied normalized spectral clustering to the neural STRFs to obtain nine clusters. Next, we computed the eMTF for each cluster, extracted isoline contours at the 65% level, and overlaid these curves on the eMTF of the model STRFs for 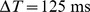. These results are shown in Figure 6 and illustrate the overlap between the model and neural data, particularly at the “edges” of the neural STRF modulations. While the overlap is not complete, it is clear that the modulation spectra of each ensemble are not disjoint. Moreover, the model eMTF suggests a general ensemble sensitivity to relatively fast modulations; this point is explored further in a later section (“Emergent STRFs capture spectro-temporal modulation statistics of stimulus”).

To better characterize the relationship between the neural and model data, we employed a statistical comparison of the distribution of the two datasets. If the models truly generated STRFs similar to those in physiological studies, then one might expect a nearest-neighbor (NN) similarity distribution akin to one derived from the neural ensemble we considered. We computed the symmetric KL-divergence between each of the model and within-physiology NN similarity distributions (shown in Supplemental Figure 2). We found that the sustained-response (presented here) and sustained-shape (presented later in this paper) distributions had KL divergences of 0.80 and 0.85, respectively, whereas the sparse distribution had a KL distance of 1.05. KL typically measures the expected number of bits required to code samples from one distribution using codes from the other. While these numbers are difficult to assess in absolute terms, they give a sense of how the different model optimizations and constraints compare to each other. These numbers reveal that the sustained ensembles are similarly comparable to the physiology, whereas the sparse ensemble has a somewhat worse match. Of course, caution must be taken with these numbers because the set of neural STRFs we analyzed represent only a subset of mappings that likely exist in central auditory areas.

**Figure 7:**
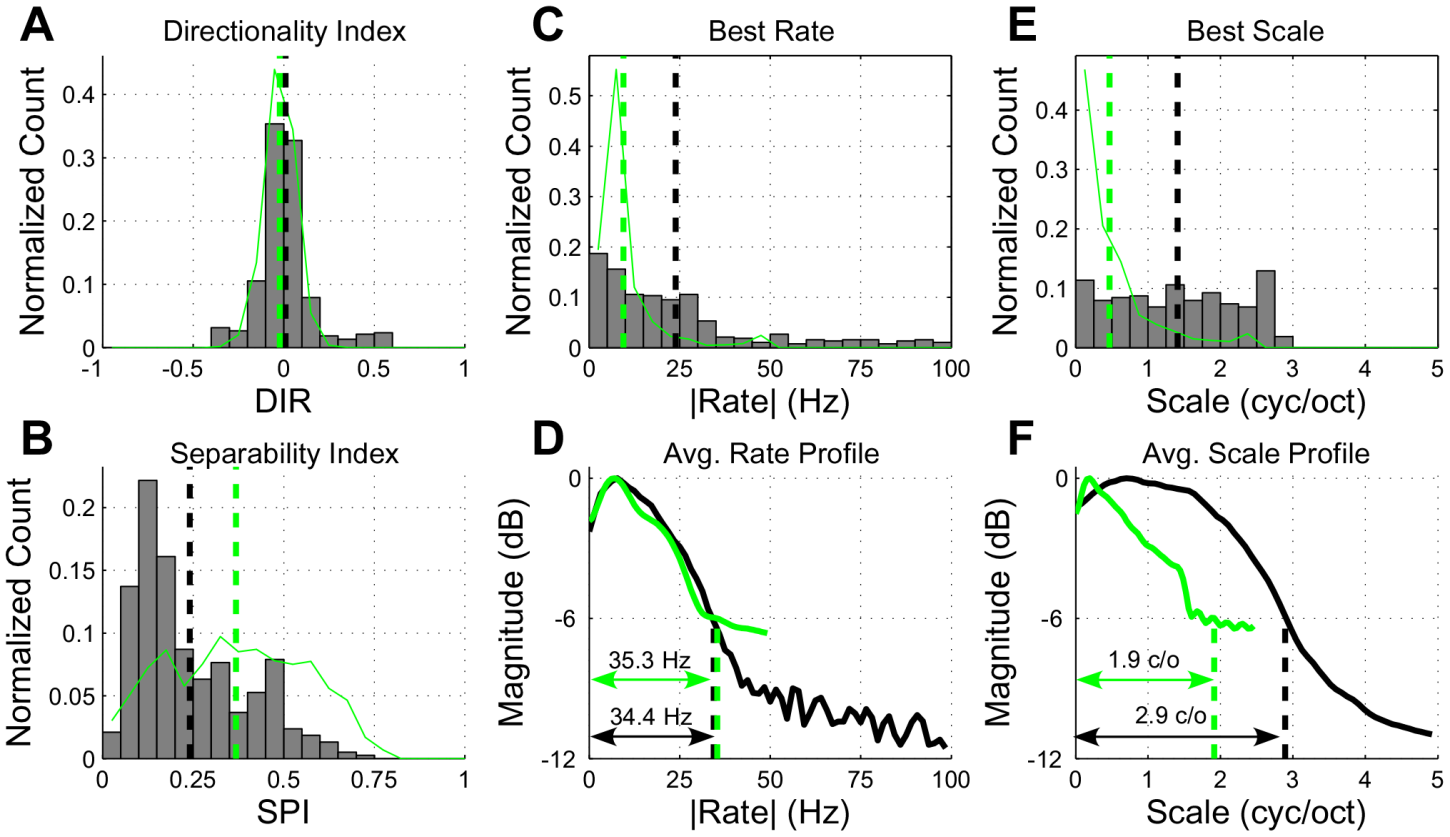
Ensemble analysis of STRFs learned under the sustained objective function for 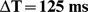
 **.** In panels (A), (B), (C) and (E), the histograms show the distribution of model parameters whereas the thin green lines show the distribution of the physiological data. The black and green dashed vertical lines show population means for the model and neural data, respectively. In panels (D) and (F), the black and green lines correspond to the model and neural STRFs, respectively, with the dashed lines indicating 6-dB upper cutoff frequencies. Refer to the text for more details.

Next, we measured a variety of parameters from the neural and model STRFs (for 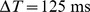) that more fully characterized the extent of spectro-temporal coverage and modulation sensitivity of the ensembles (see Methods), the results of which are summarized in Figure 7.

Based on the distribution of directionality indices, shown in panel (A), we observe that the model STRFs are largely symmetric, with the majority of neurons having no preference for upward or downward moving input stimuli (mean
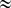
0). As indicated by the tails of this distribution, however, a subset of neurons have a strong directional preference. This agrees with the neural STRFs, and similar observations have been made in MGB and primary auditory cortex of cats by Miller *et al.*, as well as in measurements by Depireux *et al.* from primary auditory cortex of ferrets. Furthermore, panel (B) illustrates that a large number of model STRFs are fairly separable, with a peak in the separability index (SPI) distribution around 0.10 and an average value of 0.26. This trend aligns with values reported in the literature by Depireux *et al.* in measurements from ferret auditory cortex (mean of approx. 0.25). However, it is worth noting that this low level of separability is not uniformly reported across physiological studies of receptive field of mammalian auditory cortex. For instance, the physiological data analyzed in the current study (examples of which are shown in Figure 5) do yield a higher average SPI (mean = 0.37).

The temporal modulation statistics of the model STRFs, as quantified by best rate (BR), also align generally with results reported from mammalian thalamus and cortex. In panel (C) we observe a broad, bandpass distribution of best rates, with an average of 23.9 Hz. Reported physiological results from Miller *et al.* show similarly broad ranges of temporal tuning with preferences around 16 Hz and 30 Hz range for cortex and thalamus, respectively. The neural STRFs we analyzed show a somewhat slower tuning, with an average BR of 9.5 Hz. Furthermore, in panel (D), we computed the normalized average rate profile from the model STRFs. We observe a peak at 7.8 Hz, with an upper 6-dB cutoff of 34.4 Hz. Here we find a close overlap with the rate profile computed from the neural STRFs as well as with average profile results as reported by Miller *et al.* (peak at 12.8 Hz; upper 6-dB cutoff at 37.4 Hz).

The spectral modulation statistics of the model STRFs, as quantified by best scale, are generally faster than those reported from studies of thalamic and cortical nuclei. The distribution of best scales shown in panel (E) is bandpass with a wide range of slow to fast spectral coverage, with an average tuning of 1.40 cyc/oct. The neural STRFs, in contrast, are tuned to much slower scales (mean = 0.47 cyc/oct). Similarly, results from Miller *et al.* in MGB indicate a generally slower tuning (0.58 cyc/oct), whereas measurements from cortical neurons, while having a similarly wide range of tunings as with the model, indicate a slower average value of 0.46 cyc/oct and an upper cutoff of approx. 2 cyc/oct.

Finally, the ensemble average scale profile, shown in panel (F), is bandpass and exhibits a peak at 0.7 cyc/oct with an upper 6-dB cutoff of 2.9 cyc/oct. The neural STRFs, however, are much slower with peak at 0.2 cyc/oct and an upper cutoff of 1.9 cyc/oct. This is similar to observations from MGB by Miller *et al.*, where they reported that the ensemble average scale profile is generally low-pass, with average scale profile peaks and upper 6-dB cutoffs at 0 cyc/oct and 1.3 cyc/oct, respectively, with similar observations in cortex.

In summary, while we cannot map the emergent STRFs to any exact synapse, they nevertheless reflect the general processing characteristics of various stations along in the central auditory pathway. There is good alignment with the neural STRFs and reported results in mammalian MGB and primary auditory cortex with respect to directional sensitivity and spectro-temporal separability. The temporal modulation statistics of the emergent sustained STRFs appear to be most similar to those measured from thalamus and cortex. Furthermore, the model STRFs are generally faster with regard to spectral modulations than those measured from thalamus and cortex.

### Emergence of a sparse population code

To explore the relationship between STRFs optimized to promote sustained responses and those that explicitly maximize population sparsity, we compared the average responses of the sustained ensemble for 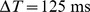 with the sparse ensemble. Specifically, we used the converged STRFs to analyze a held-out set of natural stimuli, computed a histogram of the population responses at each time, and computed the average histogram across the entire test input (see Methods). Since the sparse ensemble was optimized to yield a highly kurtotic firing rate distribution, it was of interest to examine the shape of the distribution when promoting sustained responses.

**Figure 8:**
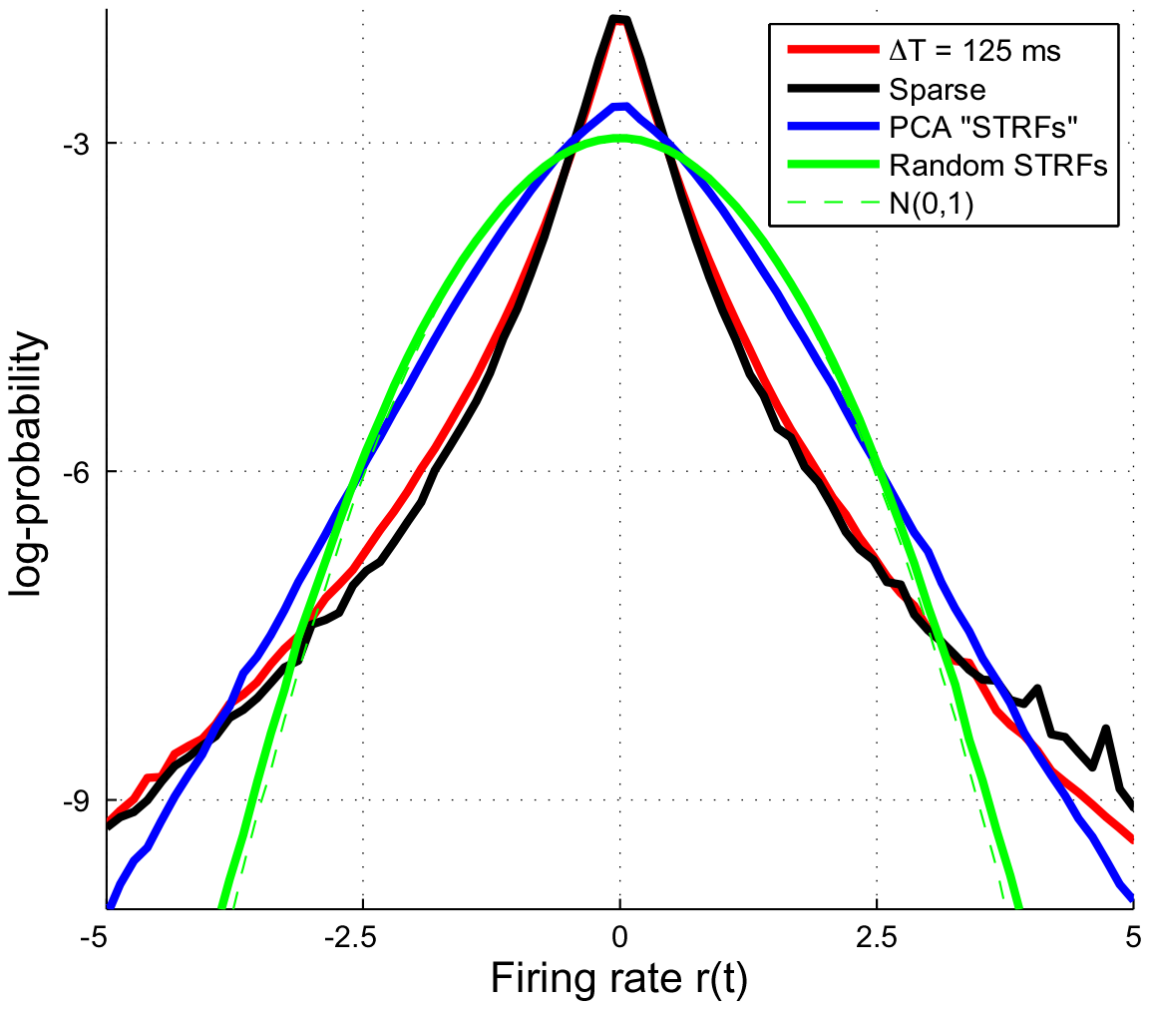
Average population response histograms for STRFs learned under the sustained and sparse objectives subject to response constraints.

Results comparing the average histograms of sustained versus sparse responses is shown in Figure 8, with log-probabilities shown on the vertical axis to emphasize differences between the tails of the distributions. The main observation is that both the sustained and sparse ensembles have distributions that have long tails and are are highly peaked around a firing rate of zero. For reference, we show the average histograms obtained by filtering the stimulus through the first 400 principal components of the stimulus (see Supplemental Figure 3) as well as through a set of 400 random STRFs; a zero-mean, unit variance Gaussian distribution is also shown. Therefore, despite promoting temporally persistent responses, the sustained responses yield a population response that is not altogether different from an ensemble that explicitly maximizes kurtosis. Interestingly, this observation was also made by Berkes and Wiscott in the context of complex cell processing in primary visual cortex (see Sec. 6 of [33]).

### Emergent STRFs capture spectro-temporal modulation statistics of stimulus

**Figure 9:**
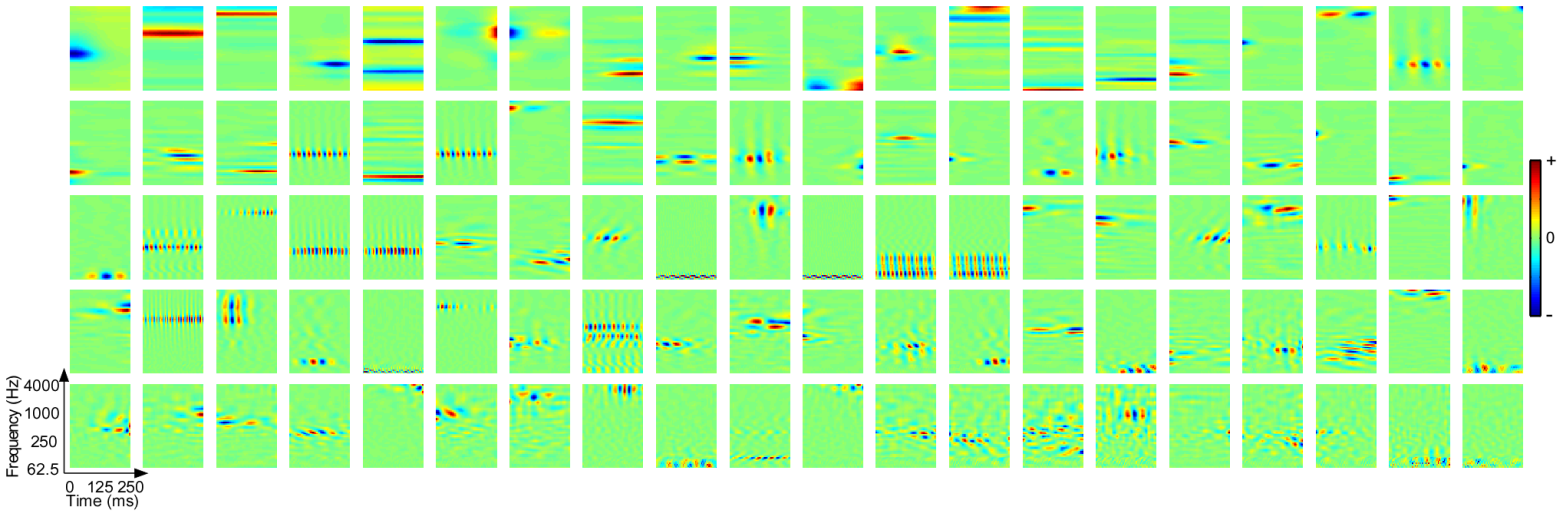
Examples of STRFs learned under the sustained objective function (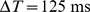) subject to orthonormality constraints on the shapes of the filters. The examples shown here were drawn at random from an ensemble of 400 neurons, and the STRFs are shown in order of decreasing contribution to the overall objective function. Each spectro-temporal patch spans 0–250 ms in time and 62.5–4000 Hz in frequency. For these examples the dynamic range of the STRFs was compressed using a 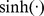 nonlinearity.

Finally, we sought to explore the consequences of relaxing the constraint that the responses be mutually uncorrelated. Rather than directly constrain the *responses*, we considered constraints to the *shapes* of the model STRFs. This was achieved by solving
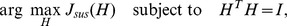
i.e., we require the STRFs to form an orthonormal basis. So long as the stimuli are bounded, this set of constraints meets our requirements that (1) the output of the STRFs be bounded and (2) we minimize redundancy in the learned ensemble. As before, the optimization is described in the Methods. We consider an ensemble size of 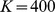 STRFs initialized at random. Examples of shape-constrained STRFs that optimize the sustained objective function for 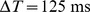 are shown in Figure 9. Again, we observe STRFs that are bandpass, localized, oriented, and sensitive to a variety of spectral and temporal input. However, there was an apparent difference between the speed of the spectro-temporal modulations and those from STRFs learned subject to the response constraints.

It is well known that natural sound ensembles are composed largely of slow spectro-temporal modulations [29], [30], [34]. However, the emergent STRFs learned subject to *response* constraints appear to be tuned to relatively *fast* spectral and temporal modulations, whereas the STRFs learned subject to *shape* constraints appear to have a broader tuning. To further examine how both sets of constraints jointly capture and are related to the spectro-temporal modulations observed in stimulus, we compared the average 2D modulation profile of the stimulus to the eMTFs derived from both sets of constraints.

**Figure 10:**
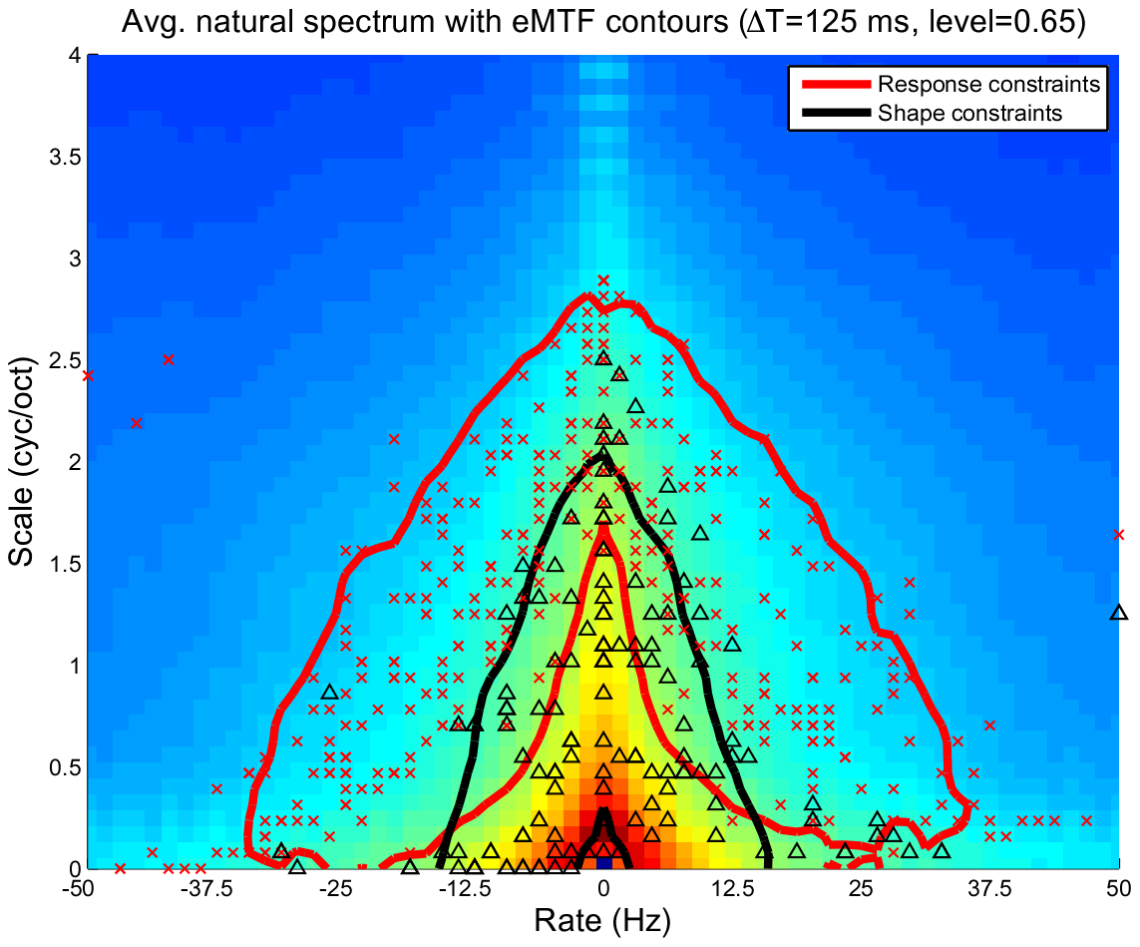
Spectro-temporal modulations in the stimulus are fully captured by STRFs that promote sustained responses subject to response and shape constraints. Here, the average MTF of the stimulus is overlaid with contours (at the 65% level) of the ensemble MTFs for both constraints for 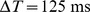. For each ensemble we also show the constellations for best rate vs. best scale (marked by ‘
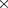
’ and ‘
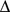
’ for response and shape constraints, respectively). For the response constraints, we show the contour line and BR/BS constellations for STRFs that contribute to 99% of the objective function.

An interesting view of how the emergent STRFs capture the spectro-temporal modulations of the stimulus is illustrated in Figure 10 for 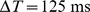. Shown is the average 2D modulation profile of the stimulus overlaid with a single isoline contour (at the 65% level) of the eMTFs learned subject to response (thick red lines) and shape constraints (thick black lines). We also show the constellation of BR versus BS for each ensemble (indicated by ‘
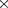
’ and ‘
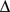
’ for response and shape constraints, respectively). As implied by the contours, the response constraints yield STRFs that follow the spectro-temporal “edge” of the stimulus, while the shape constraints explicitly capture most of the “slowness” of the stimulus. As mentioned previously, the response constraints effectively force the temporal response of the sustained ensemble to be sparse, which consequently results in highly selective STRFs that tend to be tuned to fast modulations. Nevertheless, they implicitly capture the spectro-temporal extent of the stimulus. Moreover, since the shape constraints effectively force the STRFs to form a basis that spans the input space, this results in neurons that explicitly capture the slow modulations of the stimulus. Similar observations were made across the range of 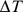, and for each case it was clear that the spectro-temporal modulations of the stimulus are fully captured by the combination of both sets of constraints.

## Discussion

In this paper, we considered a framework for studying how choice of a sustained firing versus sparse coding objective affects the shapes of model spectro-temporal receptive fields in central auditory areas. The sparse coding objective considered here, namely that of maximizing population kurtosis, yields STRFs that are mostly noisy. Those that do converge are generally highly localized. In contrast, enforcing the sustained firing objective subject to the same response constraints yields richly structured ensembles of STRFs whose population diversity varies smoothly with the correlation interval 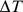. Of course, the observed structural variations are necessarily biased due to construction of the stimulus. Nevertheless, this diversity, as revealed by the results of the unsupervised clustering, paired with the responses of the most persistent STRFs, supports the notion that sustained neural firings are preferred in the range of timescales predominant in natural sounds. While we do not necessarily attribute the emergent sustained STRFs to any particular synapse in the auditory pathway, we instead note that the observed filters exhibit general similarities to physiological observations made in auditory thalamus and cortex.

We also observed that enforcing the sustained firing objective with response constraints yields an ensemble firing rate distribution that is similar, on average, to one where population sparsity was explicitly enforced. This supports the proposal that the two coding objectives are not necessarily at odds, and that in some sense a sustained firing objective yields “sparsity for free.” Of course, the sustained firing and sparse coding objectives could be quantified in many different ways (see, e.g., Hashimoto [35] and Carlson *et al.* [11]), but the present study is a promising step in understanding their relationship in the central auditory system from a computational perspective.

Finally, to explore the consequences of relaxing the constraint that the responses be mutually uncorrelated, we explored an alternative set of orthonormality constraints on the sustained firing objective. While still minimizing a notion of redundancy, we observed that the emergent ensembles are generally slower, potentially better capturing the slow spectro-temporal modulations known to be present in natural sounds. This experiment further demonstrated the utility of the considered framework for directly addressing questions about coding schemes and various sets of constraints in representing sound in central auditory areas.

### Emergence of a discriminative spectro-temporal representation for natural sounds

The combination of shape and response constraints on the sustained objective function yield STRF ensembles that appear to jointly capture the full range of spectro-temporal modulations in the stimulus. However, the distinct differences in MTF coverage illustrate the tradeoff between redundancy and efficiency in sensory representations. In particular, the shape constraints yield STRFs that are somewhat akin to the first few principal components of the stimulus (see Supplemental Figure 3). This is not surprising given that the objective function defines a notion of variance of linear projections, the component vectors of which are constrained to form an orthonormal basis. However, since the responses are not strictly enforced to be uncorrelated, orthonormality imposed on the filter shapes does not necessarily reduce redundancy in the resulting neural responses.

In contrast, the response constraints yield STRFs that are highly selective to the input and are thus comparatively “fast” in the modulation domain. This representation can be thought of as more efficient since at any given time only a few neurons have a large response. However, while the shapes of individual STRFs fail to explicitly capture the slow spectro-temporal modulations predominant in natural sounds, it instead appears that the ensemble MTF of the response-constrained STRFs collectively forms a contour around the high-energy modulations of the stimulus that implicitly capture its spectro-temporal extent.

Is this contouring of the average modulation spectrum of natural sounds something performed by the auditory system? The neural STRFs we considered certainly had an eMTF that reflects a tuning to slower modulations near the MTF origin. However, there is some evidence that the auditory system uses an “edge”-sensitive, discriminative modulation profile for analyzing sound. Woolley *et al.* [36], in an avian study, showed that the eMTF of neurons from Field L (the avian A1 analog) has a bandpass temporal modulation profile (at low scales) that facilitates a discriminative tuning of temporal modulations among classes of natural sounds. Nagel and Doupe [37] have also shown examples of avian Field L STRFs that orient themselves near the spectro-temporal “edge” of the stimulus space. Moreover, Rodriguez *et al.* [38], in a study of mammalian IC neurons, showed that neural bandwidths can scale to better capture fast, but less frequently occurring, modulations. In light of these observations, the modulation profiles observed from the sustained STRFs for both response and shape constraints are consistent with the notion that the auditory system makes an explicit effort to capture all modulations present in natural sounds: fast, feature-selective, and consequently *discriminative* modulations, as well as frequently occurring slow modulations.

### A neural code for sensory processing

The notion that sustained neural firings form part of the neural representation of sensory systems is not limited exclusively to the auditory modality. In fact, the sustained firing objective considered in this paper is related to a broad class of sensory coding strategies referred to collectively under the *temporal slowness hypothesis*. This concept proposes that the responses of sensory neurons reflect the time-course of the information-bearing components of the stimulus—which are often much slower with respect to the fast variations observed in the stimulus—and may therefore reflect invariant aspects of the sensory objects in the environment. Examples of early neural network models exploring slowness as a learning principle were considered by Földiák [39], Mitchison [40], and Becker [41]. More recently, a number of computational studies, particularly in vision, have established slowness as a general sensory coding strategy and have revealed relationships with a number of general machine learning techniques. Here we outline the connections between the sustained firing criterion considered in this study and previous work.

Our definition of the sustained firing objective, 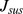, was adapted from a notion of temporal stability proposed by Hurri and Hyvärinen termed *temporal response strength correlation* (TRSC) [18]. This study considered modeling of simple cells in primary visual cortex, and their objective function was defined as
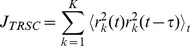
(3)for a single fixed 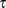. By maximizing 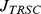 subject to the decorrelation constraints 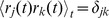, they showed the emergence of spatial receptive fields similar to those observed in simple cells in primary visual cortex. It is clear that the objective functions 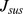 and 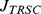 are equivalent for a single time step, but the main difference between the two is that we sought to enforce temporal stability over a time *interval* 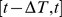, rather than between two *distinct* times 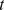 and 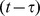. Interestingly, optimization of the TRSC objective was shown by Hyvärinen to yield a solution to the blind source separation problem [42], suggesting perhaps that in the auditory domain, such a criterion may underlie separation of overlapping acoustic sources.

The sustained firing objective is also related to a well-known model of temporal slowness known as *slow feature analysis* (SFA) [20]. The computational goal of SFA is to find a mapping of an input that extracts the slow, and presumably more invariant, information in the stimulus. Briefly, for an input 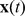, linear SFA finds mappings 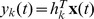 that minimize
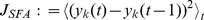
(4)subject to 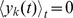, 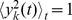, and 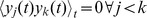. Note that the input 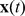 is not necessarily the raw stimulus but could represent a non-linear expansion of the input, akin to applying a kernel function in a support vector machine [43]. Therefore, SFA finds a mapping of the input that varies little over time and whose outputs are bounded and mutually uncorrelated. In the visual domain, Berkes and Wiskott found that SFA could explain a variety of complex cell phenomena in primary visual cortex such as the emergence of Gabor-like receptive fields, phase invariance, various forms of inhibition, and directional sensitivity [33]. Similar to our study, they also found the emergence of a sparse population code based on SFA. More importantly, however, they established a link between SFA at the level of complex cells and 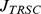, which in turn links to the sustained firing objective 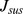 explored in our study. Specifically, they showed that when a complex cell output is expressed as a quadratic form 
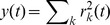
 [35], [44], the SFA objective could be written as
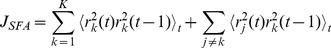
(5)which is equivalent to maximizing 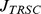 (and thus 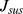 for a single time-step) plus cross-correlation terms. As noted by Berkes and Wiskott, this relationship suggests that sustained firing rates at the level of simple cells are modulated as part of a hierarchical cortical processing scheme in primary visual cortex. Given the increasing understanding of such hierarchical circuits in the auditory system [45], the possibility that sustained firing rates are varied as part of a higher-order processing strategy in primary auditory areas is an exciting prospect worth further exploration.

Other important relationships exist between SFA and a number of general machine learning principles. Blaschke *et al.* [46] established a relationship between SFA and independent component analysis, a widely used method for blind source separation (see, e.g., [47]). Klampfl and Maass [48] showed that under certain slowness assumptions about the underlying class labels in observed data, SFA finds a discriminative projection of the input similar to Fisher's linear discriminant. Furthermore, SFA has links to methods for nonlinear dimensionality reduction: Creutzig and Sprekeler [49] described the link between SFA and the information bottleneck whereas Sprekeler [50] showed a connection between SFA and Laplacian eigenmaps.

In summary, the temporal slowness hypothesis forms a sound basis for learning a representation from data with rich temporal structure. Slowness as a learning principle has also been shown to explain the emergence of simple and complex cell properties in primary visual cortex. As described above, the sustained firing principle considered in this paper has fundamental links to SFA, which in turn is related to a number of general machine learning strategies. To the best of our knowledge, ours is the first thorough study that establishes a link between the temporal slowness hypothesis and an emergent spectro-temporal representation of sound in central auditory areas.

### Implications for automated sound processing systems

The ensemble modulation coverage results are particularly interesting since it is widely thought that “slow” spectro-temporal modulations carry much of the message-bearing information for human speech perception. Furthermore, it is known in the speech processing community that features that capture slow temporal [51] and joint spectro-temporal modulations [52], [53] are important for noise-robust automatic speech recognition. The observed contouring effect resulting from the sustained firing criterion may thus reflect a mechanism to detect the spectro-temporal “edges” of the message-bearing components of the stimulus, and possibly contribute to a noise-robust representation of sound. We have recently considered this principle and have demonstrated that 2D bandpass filters derived from eMTF contours learned from a speech-only stimulus yield state-of-the-art noise-robust acoustic features for automatic speech recognition [54]. Moreover, it is possible that the contour level may be chosen adaptively as a function of ambient signal-to-noise ratio to better capture variations in the high-energy modulations of the stimulus. Also, since the emergent STRFs capture general spectro-temporal patterns that characterize the stimulus, it is possible that ensembles of STRFs could be learned in various speech-plus-noise scenarios to perhaps better characterize noise-corrupted acoustic environments. Such hypotheses can be readily verified experimentally and may have practical impact to automated sound processing systems in noisy acoustic environments.

### Concluding remarks

Finally, the framework considered in this paper can be extended in a number of ways. For instance, to address the linearity limitation of the STRF, it is worthwhile to consider a model based on a linear-nonlinear cascade [55]. As mentioned earlier, the auditory pathway is necessarily hierarchical, and warrants consideration of hierarchical computational models. Indeed, recent physiological evidence also indicates that the representation becomes increasingly complex and nonlinear as one moves from away thalamo-recipient layers in primary auditory cortex (for a review, see [45]). Finally, a recent computational study in vision by Cadieu and Olshausen [56] proposes a hierarchical generative model that explicitly unifies notions of sparse coding and temporal stability. In particular, a two-layer network learns a sparse input representation whose activations vary smoothly over time, whereas a second layer modulates the plasticity of the first layer, resulting in a smooth time-varying basis for image sequences. One can imagine that such a framework could be extended to spectro-temporal acoustic stimuli.

## Methods

### Stimulus description and preparation

An ensemble of natural sounds comprising segments of speech, animal vocalizations, and ambient outdoor noises was assembled for use as stimuli. Two sets were generated, one for training and one for evaluating the response characteristics of the STRFs. Phonetically balanced sentences read by male and female speakers were used [57]. Examples of animal vocalizations included barking dogs, bleating goats, and chattering monkeys [58]. The ambient sounds included, for example, babbling creeks and blowing wind, and other outdoor noises. The speech utterances were approximately three seconds each and comprised 50% of the stimulus. The animal vocalizations and ambient sounds formed the remaining 50% of the stimulus (25% each), were broken into three-second segments, and were windowed using a raised cosine window to avoid transient effects. Finally, segments from each class were downsampled to 8 kHz, standardized to be zero-mean and unit variance, and randomly concatenated to yield a waveform approximately three minutes in overall length, i.e., 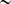
90 seconds of speech, 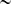
45 seconds of animal vocalizations, and 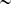
45 seconds of ambient outdoor noises.

We used a computational model of peripheral processing to account for the transformation of a monaural acoustic stimulus to a joint time-frequency representation in the auditory midbrain; this representation is referred to as an *auditory spectrogram* [59], [60]. The auditory spectrogram represents the time-varying spectral energy distribution on the (logarithmic) tonotopic axis, and accounts for the physiology of inner hair cell transduction and filtering on the auditory nerve, enhanced frequency selectivity in the cochlear nucleus via a lateral inhibitory network, and the loss of phase locking to stimuli observed in midbrain nuclei. The specific model details have been presented previously and as such we forego a detailed description here, except to note that we sampled the log-frequency axis over six octaves with ten equally spaced channels per octave, with a short-term integration interval of 5 ms, i.e., we obtained a 60 channel spectral vector every 5 ms. An example auditory spectrogram is shown for a segment of speech in Figure 1A.

### Spectro-temporal receptive fields

To quantify the relationship between a spectro-temporal stimulus and its corresponding response in central auditory areas, we used the spectro-temporal receptive field. Such a functional characterization of a neuron is useful for identifying the components of the stimulus to which it is most sensitive. An STRF models the linear transformation of a time-varying spectro-temporal input to an instantaneous firing rate, i.e.,
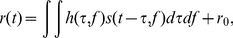
(6)where 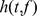 is an LTI filter that defines the STRF, 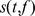 is a spectro-temporal stimulus, and 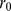 is the average firing rate. Without loss of generality, we assume 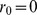. Observe that the mapping represents convolution in time and integration across all frequencies, and we can interpret the STRF as a matched filter that acts on the input auditory spectrogram.

For discrete-time signals and filters, and assuming that 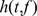 has a finite impulse response, we can express Eq. 6 compactly in vector notation as
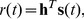
(7)where 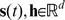 are column vectors denoting the stimulus and filter, respectively [61]. Furthermore, to express the response 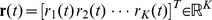 of an *ensemble* of 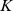 neurons, we concatenate the STRFs into a matrix 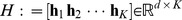 and write
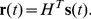
(8)


From the stimulus auditory spectrogram, we extracted 250 ms spectro-temporal segments once every 5 ms. Each segment was stacked columnwise into a vector 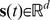 where 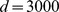 (i.e., 50 vectors/segment 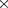
60 channels). A total of 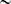
30 k spectro-temporal vectors were extracted from the stimulus. We subtracted the local mean from each segment and scaled each vector to be unit norm [18], and note that this pre-processing was also applied to the test stimulus used for evaluating the STRF response characteristics. Finally, each spectro-temporal input patch was processed by the ensemble of STRFs to yield a population response 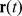. Figure 1B illustrates the procedure for obtaining stimulus vectors 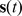 and response vector 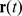.

### Optimization

To constrain the responses of the STRFs to have unit variance and be mutually uncorrelated, we first note that the individual constraints can be written as
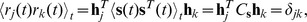
which can then be compactly expressed as an ensemble constraint
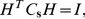
(9)where 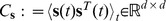 denotes the sample covariance matrix and 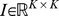 is the identity matrix. Since 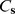 is real-symmetric, it is unitarily diagonalizable as 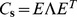, where 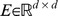 is a matrix of (columnwise) eigenvectors with corresponding eigenvalues along the diagonal of 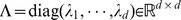. Substituting this decomposition into Eq. 9, we obtained
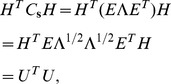
where 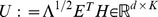. By recasting the constraints, we can rewrite the original matrix of STRFs as 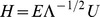 and consequently
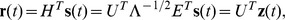
where 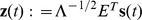 corresponds to a *whitening* of the input acoustic data, i.e., 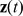 has a spherical covariance matrix. For computational efficiency, we reduced the dimensionality of the input using a subset of the principal components of the stimulus, i.e.,
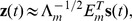
where 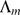 and 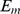, 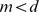, are the matrices of eigenvalues and eigenvectors, respectively, that captured 95% of the variance of the input. In this work, we found 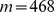. Therefore, the core problem we wished to solve is:
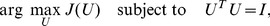
(10)where 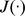 corresponded to either the sustained firing or sparse coding objective function.

To optimize this nonlinear program, we used the gradient projection method due to Rosen, the basic idea of which is as follows [62], [63]. Let 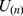 denote the 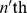 update to the matrix of (rotated and scaled) STRFs 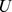, let 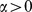 be a learning rate, and let 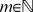 be an integer used to adjust the learning rate. Assume 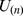 is a matrix with orthonormal columns that is a feasible solution to the problem in Eq. 10. We updated 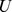 via gradient ascent as follows:
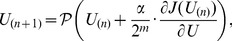
(11)where 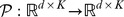 is a projection of the gradient update so that 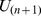 satisfies the orthonormality constraint required in Eq. 10. If the update was such that 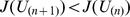, we set 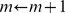 and recomputed the projected gradient update, repeating until 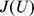 was non-decreasing. Finally, learning ceased when the relative change between 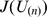 and 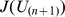 fell below a threshold 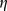 or a maximum number of iterations were reached; in our experiments, we stopped learning for 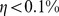 or a maximum number of 30 iterations. Upon convergence, the desired STRFs were obtained using 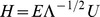. Note that for the case of the sustained firing objective, 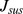 was formed from the sum of 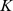 independent terms, allowing us to directly sort the emergent STRFs according to their contribution to the overall objective function; such a sorting was not possible for the sparsity objective.

Of course, the above procedure required a suitable projection 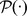, and one was derived as follows [64]. In general, for a matrix 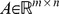, we wish to find a matrix 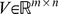 with orthonormal columns that minimizes
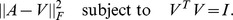
Introducing a symmetric matrix of Lagrange multipliers 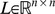, and recalling that 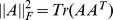, we sought to find a stationary point of the Lagrangian
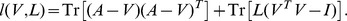
Computing the (elementwise) partial derivative of 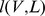 w.r.t. 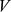 and setting it to 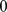 we obtained [65]

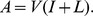
Observing that
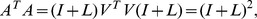
we have that
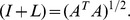
Assuming 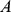 had full column rank, then an optimal orthogonal matrix that minimized 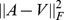 that can be used for the projection in Eq. 11 was found as
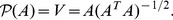
(12)


Finally, to optimize a given objective function subject to the STRFs being orthonormal, i.e., 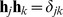, we solve
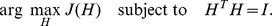
Here we can again use Rosen's projected gradient method in Eq. 11 along with the projection defined in Eq. 12, but the only difference from before is that it *does not* require pre-whitening of the stimulus.

### Characterizing individual STRFs

We first characterized the emergent STRFs based on parameters that described their individual spectro-temporal and modulation tuning.

#### Separability index

We used a measure of separability to quantify how well an STRF 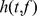 could be decomposed into a product of purely temporal and spectral functions, i.e., as 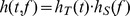 [23]. Generally speaking, by treating an STRF as a matrix 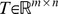, separability can be assessed by considering the singular value decomposition of 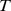:
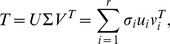
where 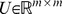 and 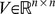 are unitary, 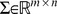 is a matrix such that the *singular values* 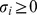 lie along the “diagonal”, and 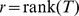. The separability index 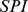 was defined as
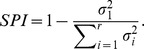
If 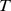 is nearly rank-1, we expect 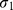 to dominate and consequently 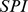 is small, indicating that 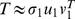, i.e., that the STRF is approximately separable as a product of only two functions. It was often the case that STRFs with a simpler structure, e.g., localized or purely spectral, had small values of 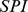. More complex STRFs, particularly those that were noisy, had larger values 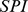 since they were poorly approximated by a low-rank decomposition.

#### Modulation transfer function

**Figure 11:**
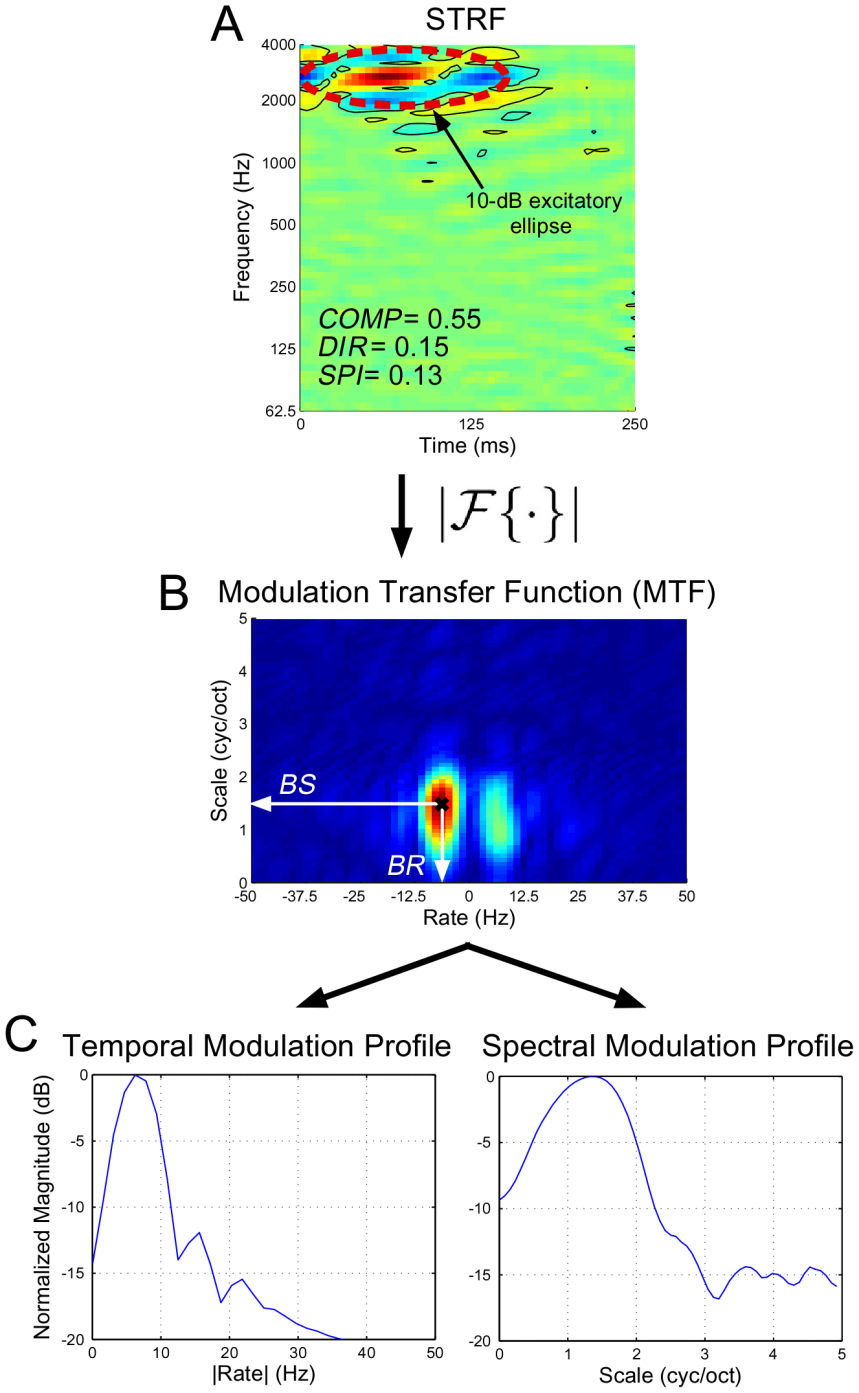
Extracting basic spectro-temporal parameters for an individual STRF. Panel (A) shows a typical STRF, with solid contour lines indicating those regions that exceed 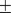 one standard deviation. The dashed red line shows the projected 10-dB ellipse from which we estimated spectral bandwidth. As indicated, the STRF is rather elongated with no strong directional preference, and the pattern is highly separable. Panel (B) shows the MTF computed from the magnitude of the 2D Fourier Transform of the STRF in (A); from here we estimate 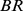 and 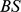. Panel (C) shows the normalized temporal and spectral modulation profiles obtained from the MTF.

To characterize spectro-temporal modulation tuning in the Fourier domain, we computed the *modulation transfer function* (MTF) of an STRF, illustrated in Figure 11B [24]. The MTF was obtained by computing the magnitude of the 2D Fourier transform of a thresholded STRF; here we set all values of the STRF that did not exceed 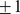 standard deviation to zero. The MTF summarizes the joint sensitivity of an STRF to temporal modulations (*rate*, in Hz) and spectral modulations (*scale*, in cyc/oct).

#### Best spectral and temporal modulation rates

We selected the peak of the MTF to estimate best rate (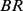) and best scale (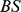). We expected that 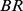 and 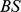 would summarize an STRF's preference for fast or slow temporal and spectral modulations.

#### Average rate and scale profiles

By folding the MTF along the 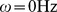 axis, we summarized the temporal and spectral modulation sensitivity of the STRF by summing along each axis, yielding rate and scale profiles; these are illustrated in Figure 11C. These profiles can also be averaged across an ensemble of neurons to yield a population rate or scale profile.

#### Directionality index

To characterize whether a neuron preferred upward vs. downward stimuli, we computed a directionality index by considering the relative difference in spectro-temporal modulation energy in the first and second quadrants in the Fourier domain. This was quantified as [23]

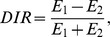
where 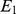 and 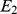 denote the energy in the first and second quadrant, respectively. By convention, 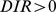 indicates a preference for *downward* moving spectro-temporal patches whereas 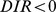 indicates a preference for *upward* moving spectro-temporal patches.

#### Compactness

To quantify a notion of compactness for an STRF, we used the *isoperimetric quotient*, which considers the ratio of the area of an ellipsoid to its perimeter, i.e.,
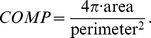
The area and perimeter were computed from the 10-dB excitatory ellipse which was derived by (1) performing a least-squares fit of a single Gaussian envelope to a thresholded STRF, (2) finding the isoline corresponding to a drop of 10-dB from the maximum of the envelope, and (3) projecting this ellipse onto the spectro-temporal plane. The compactness measure describes the degree to which the coverage of an STRF is spherical (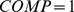) versus elongated (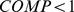), and was used for characterizing localized vs. non-localized STRFs for the purpose of grouping STRF clusters (described below).

### Characterizing STRF ensembles

Next, we considered measures that characterized a variety of ensemble-based spectro-temporal and modulation properties.

#### Ensemble modulation transfer function

By averaging the MTF obtained from each STRF, we obtained an ensemble MTF (eMTF) that characterized the average spectro-temporal modulation sensitivity of a given ensemble [24]. This representation was used to relate the average modulation tuning of an ensemble to the modulations present in the stimulus.

#### Median activation of most persistent neurons

In addition to analyzing the shapes of the emergent STRFs, we explored the ensemble firing rate characteristics of the emergent neurons. Using a held-out set of natural stimuli, we measured the activation of a neuron as the length of time a response was maintained above 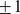 standard deviation (over time) for that particular neuron. We sorted each STRF according to its median activation time, and considered the median responses of the top 10% “most persistent” neurons for a given ensemble (as these subsets appeared to vary most across 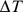). The distributions of these activations were then used to study the extent to which enforcing a sustained response was reflected in a neuron's output.

#### Average population response histogram

In order to compare distributions of population responses across ensembles, we computed averaged response histograms as follows. Upon convergence of a given ensemble, we filtered a held-out set of natural sound stimuli through the emergent STRFs to obtain a population response. At each time 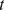, we computed a histogram of the population response, and computed the average histogram across the duration of the stimulus. These averaged histograms could then be used to compare the average population response characteristics across ensembles.

When comparing the receptive field ensembles from the sparse and sustained sets, we only included the responses of highly structured, non-noisy STRFs as determined by the clustering results outlined next. This step was necessary to keep the comparison between objective functions fair since the sparse ensemble was dominated by noisy STRFs. This inclusion criterion resulted in 115 and 347 neurons for the sparse and sustained ensembles, respectively.

For comparison, we also calculated the response histograms for stimuli filtered through the first 400 principal components of the stimulus (Supplemental Figure 3) as well as through a set of 400 random STRFs. Recall that the magnitudes of the emergent STRFs were constrained so that that their responses had unit variance over time. Accordingly, we normalized the responses of the principal components and random STRFs to also have unit variance to make a fair comparison.

### Average stimulus 2D modulation profile

To summarize the spectro-temporal modulations present in the natural sound stimulus, we averaged the magnitude of the 2D Fourier transform of 250 ms patches (non-overlapping) of the auditory spectrogram.

### Grouping canonical classes of STRFs

The optimization procedure resulted in a set of richly structured patterns that suggested the presence of a number of latent classes whose membership varied with both choice of objective function and correlation interval 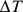. To quantify these variations, we applied the normalized spectral clustering algorithm of Ng *et al.* [66].

We defined the similarity 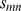 between a given pair of STRFs 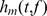 and 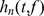 by computing the normalized 2D cross-correlation matrix for arbitrary shifts in time and frequency and selecting the maximum of the *absolute* value of this matrix, i.e.,
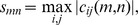
where
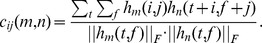
Importantly, the absolute value of the cross correlation was used here since we wished to group STRFs regardless of whether they were excitatory or inhibitory. Next, we pooled all STRFs we sought to cluster and constructed a pairwise similarity matrix 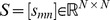. Viewing 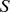 as a fully connected graph with edge weights specified by 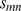, spectral clustering finds a partitioning of the graph into 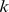 groups such that edges between groups have low similarity whereas edges within a group have high similarity.

Defining the degree matrix 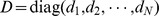 where 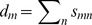 and unnormalized graph Laplacian 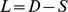, the normalized spectral clustering algorithm is as follows:

1. Compute the normalized Laplacian 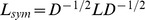.
2. Compute the first 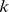 eigenvectors 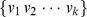 corresponding to the largest 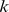 eigenvalues of 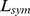.
3. Let 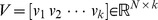 and form a matrix 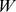 from 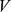 by normalizing each row to have unit Euclidean norm.
4. Denoting 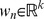 as the 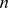
'th row of 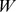, cluster the set of points 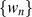 using the 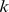-means algorithm to obtain clusters 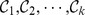.

We clustered the STRFs initially into 12 groups. While this number was necessarily an arbitrary choice, it was found to sufficiently capture variations in population diversity with 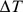. However, we found that (i) three of the resulting clusters could be reasonably labeled as *noisy*, whereas (ii) two of the resulting clusters could be reliably labeled as *localized*; merely reducing the number of initial classes did not merge the clusters, but instead blurred distinctions among the other major categories we sought to study. We interpreted *noisy* patterns as those with no obvious spectro-temporal structure and not indicative of any subset of the stimulus.

Merging of the initial 12 classes was achieved by computing the average 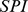 of STRFs from the initial class labels and ranking the classes in descending order. Indeed, the three *noisy* classes had the highest average 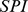 and consequently resulted in a group with average 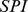 greater than 0.5. Similarly, the localized STRFs were typically highly spherical and sorting the initial clusters by 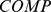 resulted in the two *localized* classes to be ranked highest. Consequently, we grouped these two clusters that had an average 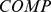 of greater than 0.69. This resulted in a final cluster count of nine classes.

### Analysis of Neural STRFs

We obtained ensembles of neural STRFs estimated using TORC [31] and speech stimuli [27], [32]. There were 2145 TORC and 793 speech STRFs, and each STRF was pre-processed to cover 110 ms in time (sampling rate = 100 Hz) and span 5 octaves in frequency (sampling rate = 5 cyc/oct). For the spectral clustering analysis, we subsampled the TORC set by randomly selecting 793 STRFs and combined them with the speech STRFs, yielding a total of 1586 STRFs in the neural data set. In this way, the neural data analysis was not biased towards one stimulus type or the other.

## Supporting Information

Figure S1
**STRFs corresponding to the top 10% “most persistent” responses for** 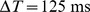.(TIF)

Figure S2 **Distributions of nearest-neighbor similarities for the model ensembles (response- and shape-constrained sustained objective vs. the sparse objective) and the neural ensemble.**
(TIF)

Figure S3 **Top 100 principal components of the natural stimulus ensemble.**
(TIF)
