# Supplementary figures and images for: Sustained Firing of Model Central Auditory Neurons Yields a Discriminative Spectro-temporal Representation for Natural Sounds
Source: PLoS Comput Biol. 2013 Mar 28;9(3):e1002982. doi: 10.1371/journal.pcbi.1002982 (PMC3610626; doi:10.1371/journal.pcbi.1002982)

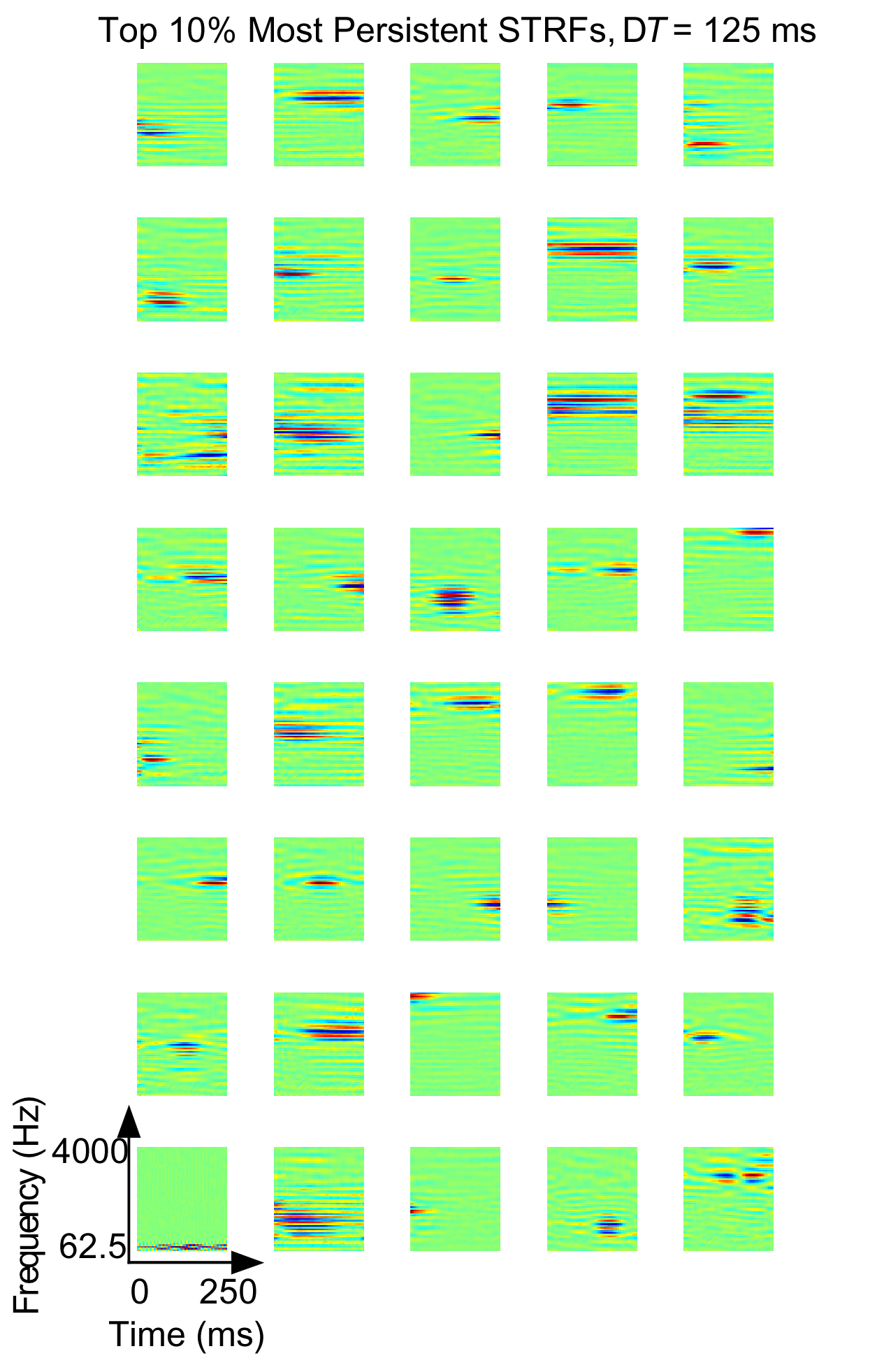

Supplement: Figure S1 — STRFs corresponding to the top 10% “most persistent” responses for . (TIF) [file pcbi.1002982.s001.tif]

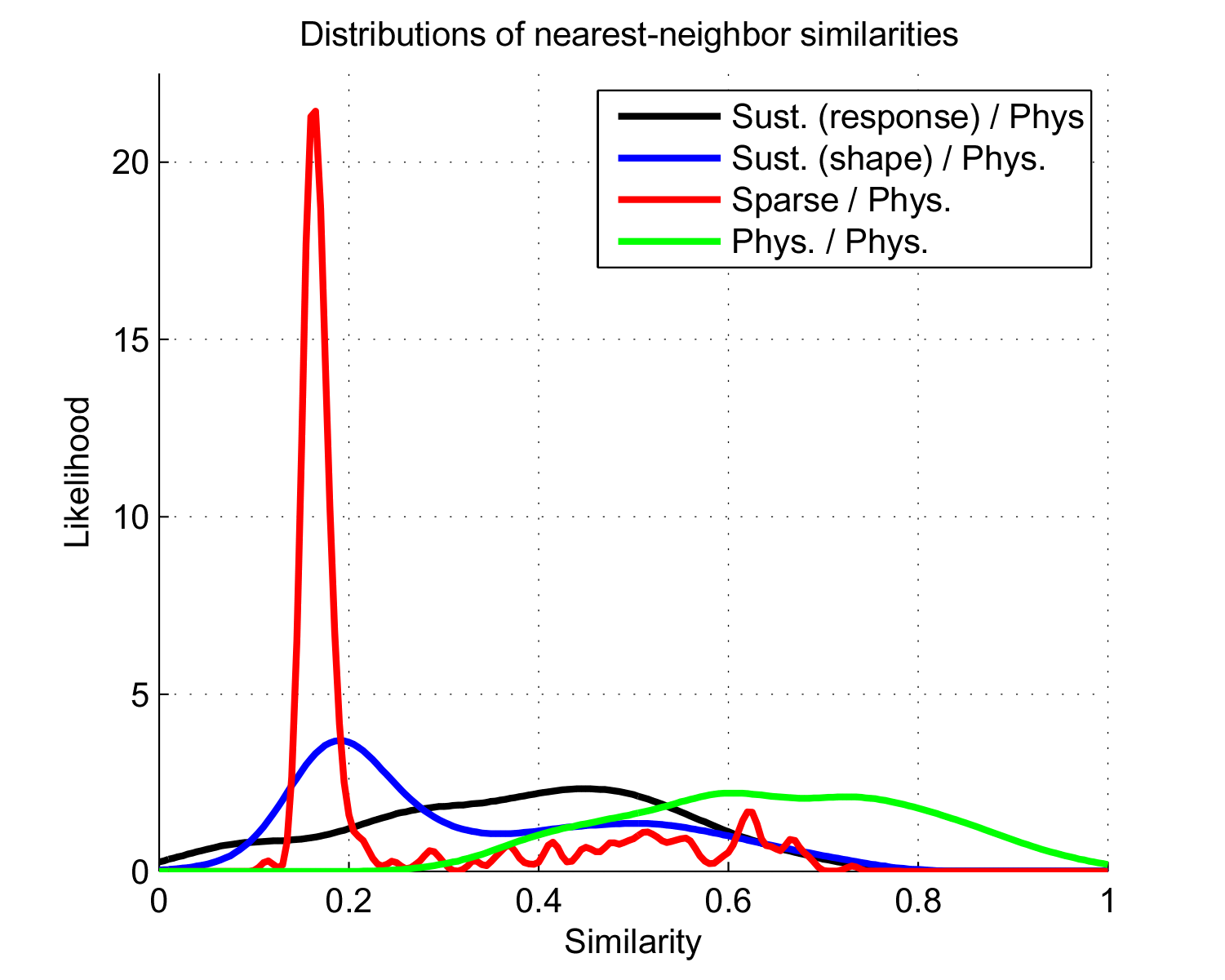

Supplement: Figure S2 — Distributions of nearest-neighbor similarities for the model ensembles (response- and shape-constrained sustained objective vs. the sparse objective) and the neural ensemble. (TIF) [file pcbi.1002982.s002.tif]

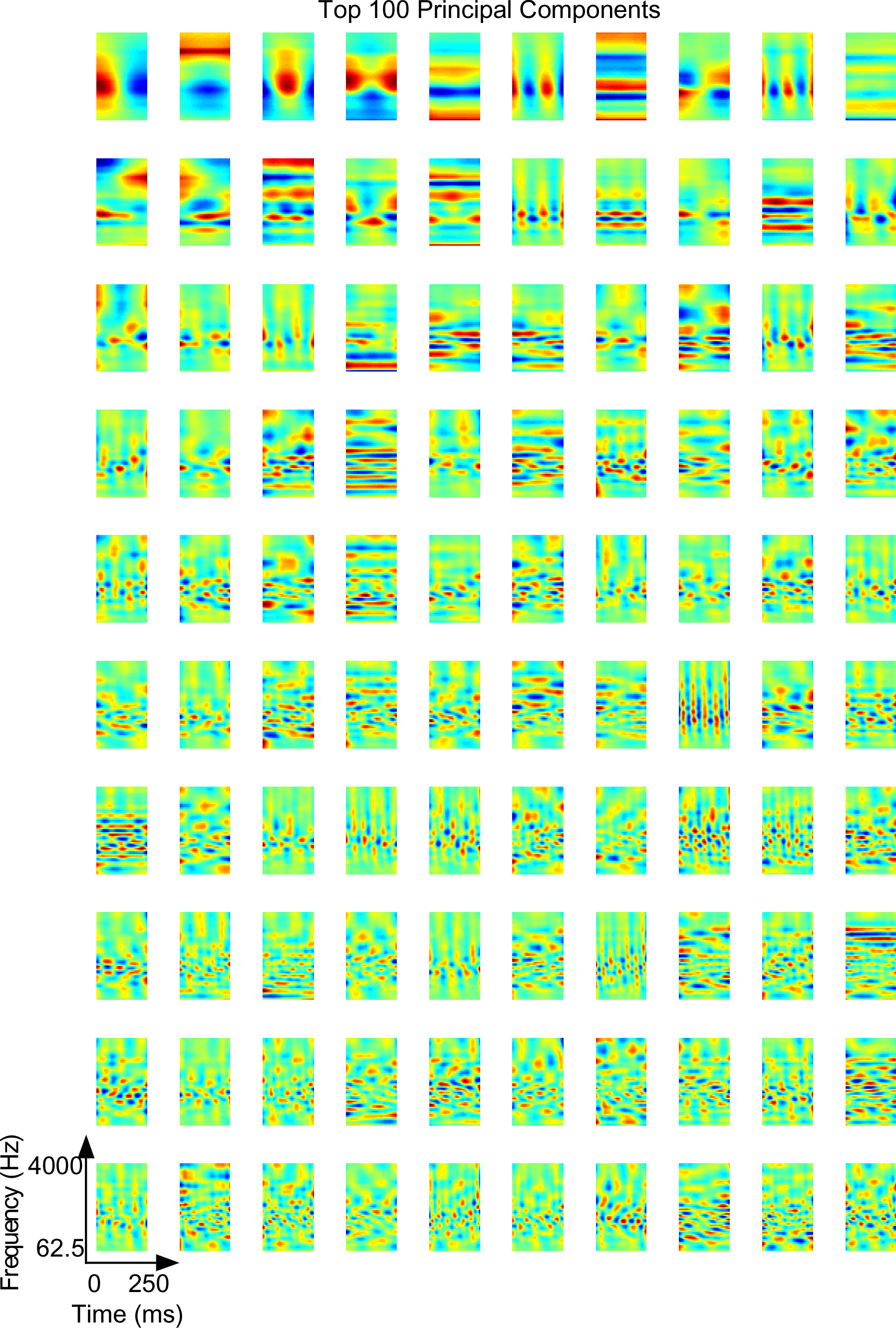

Supplement: Figure S3 — Top 100 principal components of the natural stimulus ensemble. (TIF) [file pcbi.1002982.s003.tif]
